# Pedestrian Navigation System with Trinal-IMUs for Drastic Motions

**DOI:** 10.3390/s20195570

**Published:** 2020-09-29

**Authors:** Yiming Ding, Zhi Xiong, Wanling Li, Zhiguo Cao, Zhengchun Wang

**Affiliations:** College of Automation Engineering, Nanjing University of Aeronautics and Astronautics, Nanjing 210016, China; DingYiming@nuaa.edu.cn (Y.D.); wanlingli@nuaa.edu.cn (W.L.); czg233@nuaa.edu.cn (Z.C.); wangzc@nuaa.edu.cn (Z.W.)

**Keywords:** pedestrian navigation, simultaneous localization and mapping, inertial navigation system

## Abstract

The combination of biomechanics and inertial pedestrian navigation research provides a very promising approach for pedestrian positioning in environments where Global Positioning System (GPS) signal is unavailable. However, in practical applications such as fire rescue and indoor security, the inertial sensor-based pedestrian navigation system is facing various challenges, especially the step length estimation errors and heading drift in running and sprint. In this paper, a trinal-node, including two thigh-worn inertial measurement units (IMU) and one waist-worn IMU, based simultaneous localization and occupation grid mapping method is proposed. Specifically, the gait detection and segmentation are realized by the zero-crossing detection of the difference of thighs pitch angle. A piecewise function between the step length and the probability distribution of waist horizontal acceleration is established to achieve accurate step length estimation both in regular walking and drastic motions. In addition, the simultaneous localization and mapping method based on occupancy grids, which involves the historic trajectory to improve the pedestrian’s pose estimation is introduced. The experiments show that the proposed trinal-node pedestrian inertial odometer can identify and segment each gait cycle in the walking, running, and sprint. The average step length estimation error is no more than 3.58% of the total travel distance in the motion speed from 1.23 m/s to 3.92 m/s. In combination with the proposed simultaneous localization and mapping method based on the occupancy grid, the localization error is less than 5 m in a single-story building of 2643.2 m${}^{2}$.

## 1. Introduction

The inertial pedestrian navigation system has a wide range of applications in hotspots such as firefighting, indoor security, tunnel patrols, and other fields. These practical applications typically require pedestrians to repeatedly move vigorously in the same indoor environment, such as running and sprint, rather than just regular walking. The reliable navigation information can guarantee the smooth execution of tasks and the safety of personnel in these fields. Although great progress has been made in recent years, there remain various challenging problems such as unbounded accumulative heading error and the capability in dealing with the drastic motions.

The zero-velocity update (ZUPT) algorithm has been widely applied in the field of inertial pedestrian navigation because it is suitable for the foot-mounted IMU case to compensate for traveled distance error during normal walking by introducing heuristic periodic zero velocity corrections [1]. In addition, a method fusing the navigation information of dual foot-mounted ZUPT-aided INSs was proposed [2]. This method is based on the intuition that the distance of separation between right and left foot INSs cannot be longer than a quantity known as foot-to-foot maximum separation. Shi improved the foot-to-foot maximum separation model and advanced an ellipsoid model [3].

This mechanism considers the separation constraints both in horizontal and vertical directions, which is more effective to correct the pedestrian height error. Since these methods require a long enough period of stationary time for the foot to recognize the stationary state, correct for velocity and attitude errors, and apply the separation constraints. During running and sprint, however, this stationary time is very short or even non-existent. Therefore, these methods cannot achieve the same positioning accuracy in running and sprint as in normal walking.

Another pedestrian inertial navigation method is the pedestrian dead reckoning method (PDR), which estimates the pedestrian’s step length and heading, and performs dead reckoning to track the pedestrian’s position. However, with the current state of the art of MEMS technology, the accuracy of accelerators and gyroscopes are not good enough for deriving step length and heading estimation over longer terms due to their large biases and scale factors.

Several mathematical models for indirect stride/step length estimation in the PDR algorithm. Compared with ZUPT, the indirect stride/step length estimation could restrain the accumulated error caused by the measurement noise of inertial devices after integration.

The inverted pendulum model is widely used to estimate step length in these PDR systems [4]. The geometric construction among leg length, waist vertical displacement, and step length are considered. However, the basic assumption that there always at least one foot on the ground during pedestrian movement is valid in drastic movement. The more complex models are mostly based on the statistic correlation between the step length and the acceleration of the pedestrian. For instance, the linear relation of acceleration variance, step frequency, and step length [5], and the nonlinear model between step length and maximum and minimum value of acceleration in each step [6]. These step length estimation techniques fitted the statistic features and step length in a small range, but would cause large step length error during hybrid speed motion.

As for head estimation error correction, combining both gyroscope and magnetometer inputs has yielded some success since the two sensors have complementary error characteristics—gyroscopes give poor long-term orientation, while magnetometers are subject to short-term [7,8,9]. Nevertheless, the geomagnetic field is badly disturbed by magnetic disturbances from indoor iron structures and electronic equipment. Another approach is heuristic drift reduction (HDR), assuming that many man-made walkways have straight-line features. The HDR method estimates the likelihood that the user is walking along a straight line [10,11]. If that likelihood is high, HDR applies a correction to the gyro output that would result in a reduction of drift if indeed the user was walking along a straight line. Although HDR doesn’t require landmarks to be known in advance, the basic assumption is too restrictive for complex irregular layout buildings.

Map matching could correct not only the heading estimation but also position estimation [12,13,14]. The use of environmental knowledge imposes boundary constraints on a user’s predicted motion. By using a particle filter algorithm, sub-meter tracking accuracy was achieved. However, a significantly increased amount of information needs to be provided as prior knowledge of the system for the particle filter to be effective. Robertson proposed a new Bayesian estimation approach for simultaneous mapping and localization for pedestrians based on the odometer with foot-mounted inertial sensors, called “FootSLAM”, in which the long term inertial position error is corrected efficiently using the built map of the explored area, especially when this area is revisited over time [15]. Based on FootSLAM, a series of simultaneous mapping and localization methods for pedestrians that combining the map with other information are developed. Such as Wi-SLAM [16], PlaceSLAM [17], MagSLAM [18], and SignalSLAM [19]. Moreover, Hardegger presented another kind of simultaneous mapping and localization algorithm for pedestrians using inertial sensors based on semantic landmark called ActionSLAM [20]. This method is a specific instantiation of the FastSLAM framework optimized to operate with action landmarks like open/close door, reading, brushing teeth, etc. Hardegger expanded the application of this method in different ways. [21] presented a 3D version of ActionSLAM and [22] developed a unified Bayesian framework for ActionSLAM.

The occupancy grid map is an important representation model in the mobile robot environment mapping application, proposed by Moravec and Elfes [23]. This model divides the environment where the robot is located into several neat grids and extracts the status of each grid to determine the environmental accessibility. In recent years, the FastSLAM method based on the occupancy grid using a laser range sensor has been widely applied in the field of robotic navigation. This method uses a laser range sensor to perceive the external environment and estimate the occupancy grid map of the environment. By utilizing the constructed map, the algorithm can effectively correct the positioning error of the robot. However, considering the size and cost of laser range sensors, it is not suitable for pedestrian positioning applications. For this reason, this paper proposes to construct a virtual range sensor using the inertial odometer information to sense the accessible area in the environment and construct the occupancy grid map to deal with the accumulated error of the inertial odometer.

To enhance the capability of the inertial pedestrian navigation system in dealing with the drastic motions and address the unbounded accumulated positioning error, this paper proposed a triple-node IMUs pedestrian inertial navigation system with an occupancy grid-based FastSLAM algorithm. The triple-node IMUs are composed of one waist-worn IMU and two thighs-worn IMUs. The thighs-worn IMUs are utilized to detect and segment the gait. To estimate the step length accurately for drastic motions, we introduce the horizontal acceleration probability distribution function into a step length estimation process to address the issue that the statistic feature fitting method cannot deal with drastic motion with a large speed range. Furthermore, we construct a virtual range sensor to precept the external environment and introduce it into an occupancy grid-based FastSLAM algorithm to correct the long-term position error of the inertial odometer.

This paper is organized as follows: In Section 2, the wearable trinal-IMUs pedestrian navigation system is described. The inertial simultaneous localization and occupancy grid mapping is discussed in Section 3. Analysis of both experiments and results is given in Section 4. Finally, the conclusions and future work are summarized in Section 5.

## 2. Triple-Node IMUs Pedestrian Navigation System

**Figure 1:**
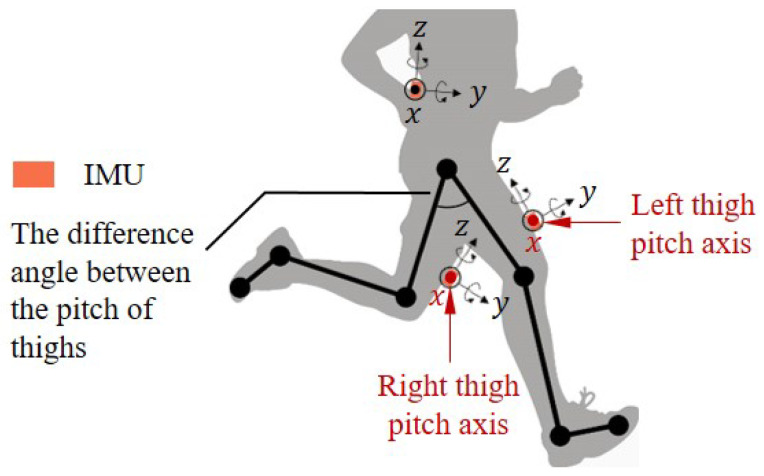
Illustration of the triple-node IMUs pedestrian navigation system sensors deployment and the lower limbs model.

This section presents the step detection, step length estimation, and heading estimation algorithms in the proposed trinal-IMUs pedestrian navigation system. In this work, three body locations are waist and thighs. We refer to one waist-worn IMU and the two thigh-worn IMUs. Each IMU is comprised of one 3D-accelerator and one gyroscope, as shown in Figure 1.

### 2.1. Step Detection

Most of the human drastic movements are driven by thighs with the strong periodicity, especially for running and jumping. In order to describe the periodicity feature of thighs changes, a lower limb model is constructed, as shown in Figure 1. The model represents the human leg as a set of joints and links. The joints are chosen to be the hip, knee, ankle, and metatarsal. The links which connected two consecutive joints are the thigh, shank and foot.

The thigh-worn IMUs are not parallel to the femurs due to the presence of muscle tissue in the thighs. To eliminate the deviation between thigh-worn IMU and thigh frame, a model alignment is required in addition to the initial alignment of the inertial navigation system.

The initial alignment process requires the pedestrian to stand still before the navigation begins. When INS is stationary with respect to the ground, the accelerometer measurements only include gravity and the gravity direction coincides with the *z*-axis of *n*-frame, so that the attitude matrix can be obtained by the following equation:

(1) $$f_{ib_{0}}^{b_{0}}=C_{n}^{b_{0}}g^{n}$$

where $b_{0}$ is the initial body frame, *n* is the local navigation frame (ENU), $f_{ib_{0}}^{b_{0}}$ is the accelerometer measurements, $C_{n}^{b_{0}}$ is the initial attitude matrix, $g^{n}={\left[0,0,g\right]}^{T}$ is the gravity vector with reference to *n*-frame.

The purpose of model alignment is to solve the attitude deviation matrix of the body frame *b* with reference to the thigh frame Th to eliminate the effect of installation error on step detection. Since the thigh-worn IMU is fixed to the thigh during the navigation process, meaning the IMU is stationary relative to the thigh, the attitude deviation matrix is constant:

(2) $$\begin{array}{cc} C_{b}^{Th} & =C_{b_{0}}^{Th_{0}}=C_{n}^{Th_{0}}{\left(C_{n}^{b_{0}}\right)}^{T}\\ \; & =\left[\begin{array}{ccc} \cos\gamma_{b_{0}} & \sin\gamma_{b_{0}}\sin\theta_{b_{0}} & -\sin\gamma_{b_{0}}\cos\theta_{b_{0}}\\ -\sin\gamma_{b_{0}} & \cos\gamma_{b_{0}}\sin\theta_{b_{0}} & -\cos\gamma_{b_{0}}\cos\theta_{b_{0}}\\ 0 & \cos\theta_{b_{0}} & \sin\theta_{b_{0}} \end{array}\right] \end{array}$$

where $C_{b}^{Th}$ is the attitude deviation matrix, ${\left[\gamma_{b_{0}},\theta_{b_{0}},\psi_{b_{0}}\right]}^{T}$ is the initial Euler angle of body frame, corresponding to the initial attitude matrix $C_{n}^{b_{0}}$.

${\left[\gamma_{Th_{0}},\theta_{Th_{0}},\psi_{Th_{0}}\right]}^{T}$ is the initial Euler angle of thigh frame and is equal to ${\left[0,\frac{\pi }{2},0\right]}^{T}$ when the pedestrian stands upright. γ, θ, and ψ represent roll, pitch, and head, respectively.

**Figure 2:**
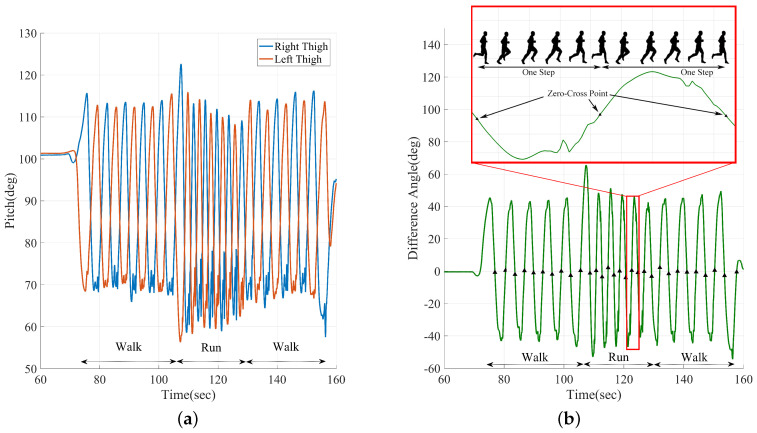
(**a**) the left thigh pitch and right thigh pitch during walking and running hybrid movement; (**b**) the difference of the left thigh pitch and right thigh pitch during walking and running hybrid movement.

After initial alignment and model alignment, the inertial odometer utilizes the difference of pitch angle between thighs to segment steps. Figure 2a presents the changes of left thigh pitch and right thigh pitch during a set of hybrid movement. It can be seen that thigh pitches change periodically and the left thigh pitch and right thigh pitch are a pair of reverse phase signals both during walking and running movement. Figure 2b presents the difference angle between the left and right thigh pitch. The difference angle changes similar to a sine wave signals in walking and running movement, and each zero-crossing point correspond to the moment when the bearing leg is perpendicular to the ground, which is the end of the last step and the beginning of the next step. Thus, the pedestrian’s steps can be separated via zero-crossing detection on the difference angle between the left and right thigh pitch, and the period between two adjacent is a complete gait circle.

**Figure 3:**
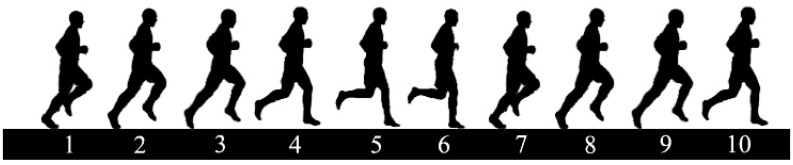
Breakdown diagram of running.

As can be seen, the zero-cross point of pitches difference correspond to step 6 of one running action breakdown, and the pitches difference zero-crossing point correspond to step 6 in Figure 3. In such circumstance, the positive value corresponds to the gait with left foot in front, and negative value corresponds to the gait with right foot in front. According to the phase characteristics of $\theta_{ThL}$ and $\theta_{ThR}$, corresponding to a step of pedestrian walking and running, the combination of zero-crossing detection method and maximum detection method can detect and segment steps well. Therefore, the difference of $\theta_{ThL}$ and $\theta_{ThR}$, $\Delta \theta_{Th}=\theta_{ThL}-\theta_{ThR}$, is used to detect and segment walking and running gait as follows:

(3) $$SD=\left\{\begin{array}{cc} 1 & if\left.\left(\Delta \theta_{Th}\left(t-1\right)\times \Delta \theta_{Th}\left(t\right)<0\right)\&\left(D_{\Delta \theta }>T_{d}\right)\&\left(\max\left(\Delta \theta_{Th}\right)>T_{max}\right)\right)\\ 0 & \;\mathrm{otherwise}\; \end{array}\right.$$

where SD is the flag bit of gait detection, $D_{\Delta \theta }$ represents the interval between two adjacent gait. $\max\left(\Delta \theta_{Th}\right)$ is the maximum of $\Delta \theta_{Th}$ within a gait movement. $T_{d}$ and $T_{max}$ is the threshold of $D_{\Delta \theta }$ and $\Delta \theta_{Th}$ respectively. $T_{d}$ and $T_{max}$ are designed to prevent the small shaking of a pedestrian’s legs while standing from being misidentified as a gait cycle. Usually, the value of $T_{d}$ is not greater than one gait period, and the value of $T_{d}$ is greater than 0 and less than the minimum of the maximum absolute value of the difference in the pitch angle of the legs during each gait cycle. In this paper, $T_{d}=0.1s$ and $T_{max}=10^{\circ }$.

### 2.2. Step Length Estimation

For pedestrian step length estimation, the inverted pendulum model is widely used. In such model, the step length is calculated as follows:

(4) $$SL=2\times l\times sin(\theta_{OT}/2)$$

where *l* indicates the pedestrian leg length, $\theta_{OT}$ indicates the max value of angular between left and right thigh in one gait, and SL indicates the step length estimation. There are certain problems with the model regarding drastic motions. One of these is that shank and thigh of the same leg are not always parallel in one gait during running, as shown in Figure 1 The modeling error will result in a large cumulative error in position estimation. Another problem is that this model requires adjusting the leg length parameter for different users.

Another class of methods for step length estimation is the fitting of statistical features of acceleration, such as in [6] by fitting a nonlinear relationship between maximum and minimum acceleration and step length over a gait period:

(5) $$SL=K\cdot \sqrt[{4}]{A_{\max}-A_{\min}}$$

where $A_{max}$ and $A_{min}$ are the maximum and minimum acceleration during one step period and *K* is the coefficient. In addition, [5] fits the relationship between gait frequency, acceleration variance, and step length:

(6) SL=A+B·SF+C·SV

where SF is step frequency and SV is the variance of acceleration of each step. *A*, *B*, *C* are the regressive parameters. On the one hand, the vertical acceleration included in methods [5,6] introduce large errors in the estimation of the horizontal displacement. On the other hand, the maximum, minimum, and variance of the acceleration cannot completely characterize the characteristics of acceleration changes in a gait cycle. It is difficult to establish a unified relationship between step length and acceleration both in walking and drastic motion through these statistical characteristics.

The acceleration probability distribution function not only contains the maximum and minimum acceleration information but also characterizes the degree of dispersion of the data. Moreover, it contains the components of different velocities. Hence, fitting the probability distribution function and step length can achieve more accurate step length estimate, and better adaptability to various velocities. To establish a step estimation method suitable for drastic motions with a large speed range, we proposed to fit step length through a segment function of horizontal acceleration norm probability distribution of the waist-worn IMU within one gait.

Let $f_{ib}^{b}$ be the waist-worn IMU accelerator output, $C_{b}^{n}$ body-to-navigation-frame coordinate transformation matrix, and $f_{ib}^{n}$ be the specific force of body with reference to navigation frame. Then,

(7) $$f_{ib}^{n}=C_{b}^{n}f_{ib}^{b}$$

and horizontal acceleration norm $|f_{ib,h}^{n}|$ can be calculated as follows:

(8) $$|f_{ib,h}^{n}|=\sqrt{{\left(f_{ib,x}^{n}\right)}^{2}+{\left(f_{ib,y}^{n}\right)}^{2}}$$

**Figure 4:**
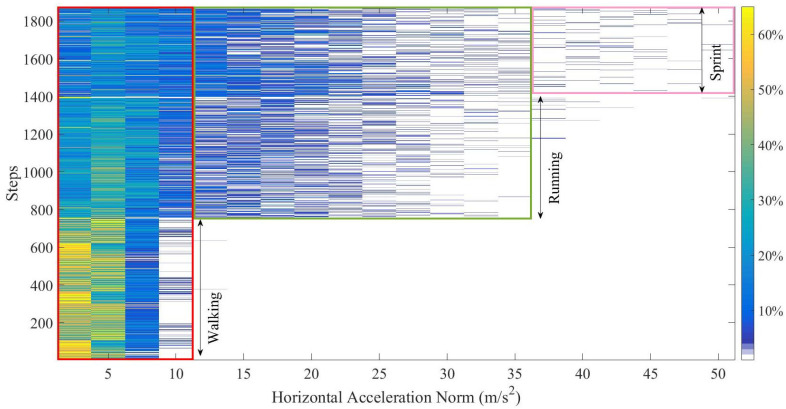
The probability distribution of horizontal acceleration among walking, running, and sprint.

Figure 4 compares the distribution of horizontal acceleration among walking, running, and sprint. Each row corresponds to one step data. In one row, the different colors correspond to the probability of horizontal acceleration norm. It can be seen that there is a clear difference of distribution in walking from other motions, which contains higher acceleration components. In addition, sprint motion has a higher percentage from 35 $m/s^{2}$ to 50 $m/s^{2}$.

Since we can record the acceleration values for each moment within one gait, and calculate its probability distribution function, $F(|f_{ib,h}^{n}|)$, the step length can be calculated by the following equation:

(9) $$SL=a_{0}F\left(x_{0}\right)+\sum_{i=1}^{K}a_{i}\left(F\left(x_{i}\right)-F\left(x_{i-1}\right)\right)$$

where $x_{i}>x_{i-1}$ and

(10) $$F\left(x\right)=P(|f_{ib,h}^{n}|<x)$$

The linear regression technique is utilized to achieve the optimal estimation of parameters $A=[a_{0},a_{1},\cdots ,a_{k}]$ in Equation (9). Assume pedestrian moves along a straight line with known length *M* times in different speeds. For the *m*-th movement, according to Equation (9),

(11) $$l_{m}=\sum_{j=1}^{N}SL_{j}=a_{0}\sum_{j=1}^{N}\left[F(x_{0,j})\right]+\sum_{i=1}^{K}a_{i}\sum_{j=1}^{N}\left[F(x_{i,j)}-F\left(x_{i-1,j}\right)\right]$$

where *l* is the straight line length, *j* is the step count number, and *N* is the number of step pedestrian takes during one movement. For brevity, we denote $F(x_{0,j})$ as $H_{0,j}$ and $F(x_{i,j)}-F\left(x_{i-1,j}\right)$ as $H_{i,j}$. Then, the optimal estimation of parameters is the vector of regression coefficients that minimizes the sum of squared residuals:

(12) $$A^{*}=\arg\min\sum_{m=1}^{M}||HA-{L||}_{2}$$

where

(13) $$\begin{array}{cc} L & ={\left[l_{1},l_{2},\cdots ,l_{M}\right]}^{T}\\ H & =\left[\begin{array}{cccc} h_{1,1} & h_{1,2} & \cdots & h_{1,K}\\ h_{2,1} & h_{2,2} & \cdots & h_{2,K}\\ \vdots & \vdots & \ddots & \vdots \\ h_{M,1} & h_{M,2} & \cdots & h_{M,K} \end{array}\right] \end{array}$$

## 3. Occupancy Grid-Based FastSLAM for Inertial Pedestrian Navigation

In general, the goal of SLAM is to estimate the pose of pedestrian *s* and the map Θ of the unknown environment. We present an inertial measurement used-only SLAM method—inertial occupancy grid-based FastSLAM. Before presenting our approach, an overview of the occupancy grid-based FastSLAM algorithm is provided.

### 3.1. Occupancy Grids-Based FastSLAM

The FastSLAM approach attempts to estimate the full SLAM posterior:

(14) $$p(s_{1:t},\Theta |z_{1:t},u_{1:t})$$

which is the distribution that represents the trajectory of pedestrian $s_{1:t}$ and the map of the environment Θ depend on the set of control inputs $u_{1:t}$ and the observations $z_{1:t}$. The Rao–Blackwellized decomposition was used to decompose the SLAM problem into a pedestrian positioning problem and an environment map construction problem with given pedestrian position:

(15) $$p\left(s_{1:t},\Theta |z_{1:t},u_{1:t}\right)=p\left(s_{1:t}|u_{1:t},z_{1:t}\right)\times \prod_{i=1}^{N}p\left(\theta_{i}|s_{1:t},z_{1:t},u_{1:t}\right)$$

where $\theta_{i}$ is the *i*-th landmark, and *N* is the number of environment features. The above decomposition shows that the a posterior probability distribution of SLAM can be decomposed into the product of the a posterior probability distribution of the pedestrian’s path and the posterior probability distribution of environment features given a known path.

The FastSLAM algorithm uses a particle filter to estimate pedestrian trajectory distribution. For each particle, the individual path-based map features are independent of each other. Therefore, in the second part of Equation (15), the conditional probability of the remaining *N* environmental map features is estimated by a bundle of binary Bayesian estimator. The conditional probability of each feature is the probability of the grid being occupied in Occupancy Grids-based FastSLAM. As a result, each particle is composed of a pedestrian pose and a map; thus, the *m*-th particle at time *t* is defined as

(16) $$X_{t}^{\left[m\right]}=\left[\begin{array}{cc} s_{t}^{\left[m\right]} & \Theta^{\left[m\right]} \end{array}\right]$$

where m=1,2,⋯n, is the index corresponding to the *m*-th particle where *n* is the total number of particles used in the particle filter, $s_{t}^{\left[m\right]}$ is the pose estimate of the *m*-th particle at time *t*, and $\Theta^{\left[m\right]}$ is the environment maintained by the *m*-th particle.

The FastSLAM algorithm consists of four basic steps: sampling new poses through the motion model, updating the environmental map, calculating the particle importance weights, and resampling based on particle weights.

#### 3.1.1. Motion Model Update

The first step of algorithm is to generate new pose for each particle by sampling from the probabilistic motion model of the pedestrian. For the *m*-th particle:

(17) $$s_{t}^{\left[m\right]}∼p\left(s_{t}|s_{t-1}^{\left[m\right]},u_{t}\right)$$

This estimate is added to a temporary set of particles, along with the path $s_{t-1}$. Under the assumption that the set of particles $s_{1:t-1}$ is distributed according to $p(s_{1:t-1}|z_{1:t-1},u_{1:t-1})$, the new particles drawn from Equation (17) are distributed according to

(18) $$p\left(s_{1:t}|z_{1:t-1},u_{1:t}\right)$$

which is referred to as the proposal distribution.

#### 3.1.2. Occupancy Grid Update

Occupancy Grid-based FastSLAM decomposes the unknown environment into a finite set of cells where each cell holds the probability that the location in the environment enclosed by the cell is occupied by an object. A solution to estimate these probability is the binary Bayes filter. In binary filter, the posterior of grid is commonly implemented as a log odds ratio in order to avoid truncation problems that arise for probabilities close to 0 or 1. The odds of grid state is defined as the ratio of the probability of this grid being occupied is divided by the probability of its negate:

(19) $$l_{t}\left(\theta_{i}\right)=\log\frac{p(\theta_{i}|s_{1:t},z_{1:t},u_{1:t})}{1-p(\theta_{i}|s_{1:t},z_{1:t},u_{1:t})}=\log\frac{p(\theta_{i}|s_{1:t},z_{1:t})}{1-p(\theta_{i}|s_{1:t},z_{1:t})}$$

and the value of posterior of grid can be recovered from the log odds form as

(20) $$p\left(\theta_{i}|s_{1:t},z_{1:t}\right)=\frac{1}{1+\exp(l\left(\theta_{i}\right))}$$

Thus, according to Bayes theory, the posterior of grid being occupied at time *t* can be estimated by involving an inverse measurement model $p(\theta_{i}|s_{t},z_{t})$:

(21) $$l_{t}\left(\theta_{i}\right)=\log\frac{p(\theta_{i}|s_{t},z_{t})}{1-p(\theta_{i}|s_{t},z_{t})}-\log\frac{p(\theta_{i})}{1-p(\theta_{i})}+l_{t-1}\left(\theta_{i}\right)$$

Here, $p(\theta_{i})$ is the prior probability of $\theta_{i}$ being occupied and is selected to be 0.5 typically.

#### 3.1.3. Importance Weight Calculation

Samples drawn from the proposal distribution are distributed according to $p\left(s_{1:t}|z_{1:t-1},u_{1:t-1}\right)$ which does not match the desired posterior $p(s_{1:t}|z_{1:t},u_{1:t})$. This difference is corrected through importance sampling. For FastSLAM, the importance weight of each particle $w_{t}^{\left[m\right]}$ is equal to the ratio of the SLAM posterior and the proposal distribution described previously:

(22) $$w_{t}^{\left[m\right]}=\frac{\;\mathrm{target}\;\mathrm{distribution}\;}{\;\mathrm{proposal}\;\mathrm{distribution}\;}=\frac{p\left(s_{1:t}^{\left[m\right]}|z_{1:t},u_{1:t},\right)}{p\left(s_{1:t}^{\left[m\right]}|z_{1:t-1},u_{1:t}\right)}=\eta p\left(z_{t}|s_{t}^{\left[m\right]},\Theta_{t-1}^{\left[m\right]}\right)$$

where $p(z_{t}|s_{t}^{\left[m\right]},\Theta_{t-1}^{\left[m\right]})$ is the probabilistic measurement model of the sensor being used by pedestrian and η is a normalizing factor—for the brevity, we simply use the normalizing factor to indicate that the weights have to be normalize to 1. The importance weight calculation procedure incorporates the sensor observations into the estimate which were not included in the pose sampling process.

#### 3.1.4. Resampling

Once the weight of particles is calculated, a new set of samples $X_{t}$ is drawn from the weighted particles with probabilities in proportion to the weight $w_{t}^{\left[m\right]}$. In such way, the new set of samples $X_{t}$ are distributed according to target distribution in Equation (14).

### 3.2. Inertial Pedestrian Occupancy Grid-Based FastSLAM Model

A key characteristic of the FastSLAM implementation is the sensing of the modality of the grid via an exteroceptive sensor. In order to minimize costs, decrease deployment effort, and remove the reliance on prior maps of the indoor surroundings, the basic idea of inertial pedestrian occupancy grid-based FastSLAM is constructing a virtual exteroceptive sensor, called ’visual range sensor’, using proprioceptive information, including the heading change and the step length estimation from the triple-node IMUs pedestrian navigation system, to track the pedestrian’s motion and build the layout of the environment.

**Figure 5:**
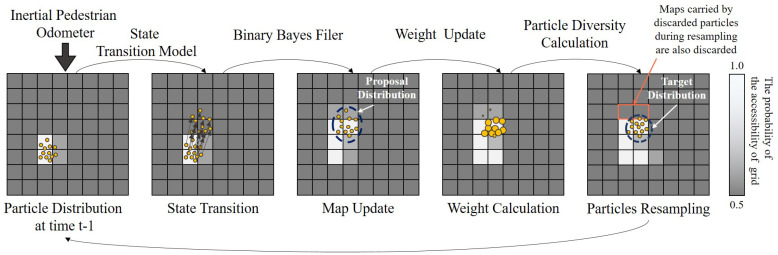
Illustration of the Inertial Pedestrian Occupancy Grid-based FastSLAM procedure.

The major steps of the inertial pedestrian occupancy grid-based FastSLAM algorithm is illustrated in Figure 5. First, the environment is divided into a set of equal size grids, and each grid holds the probability of the grid being accessible. We can sample the pedestrian’s pose at time t−1, as shown by the yellow circles. Since the pedestrian is more likely to be in accessible areas, a grid with more particles has a higher probability that it is accessible, i.e., the whiter grid in the figure. We use a beta distribution to describe this fact and the grid accessible probability is measured via a visual range sensor. The detail will be discussed in Section 3.2.2.

Once the pedestrian has moved one step, a new distribution of the pedestrian’s pose, the proposal distribution, can be calculated based on the state transition model and the pose distribution at the time t−1. Then, the probability of grid accessibility in the map is updated via binary Bayes filter. For the same reason, the grids with more particles have higher grid accessible probability, corresponding to the grids getting whiter in the figure. After the map update process, the resampling procedure proceeds, and the new particle set is the proposal distribution of the next iteration. When the pedestrian revisits the area with a built map, the particles with large error will be in the grid with smaller grid accessible probability and will be discarded after the resampling procedure. In other words, the error of the inertial odometer is corrected.

#### 3.2.1. Inertial Pedestrian Odometer and State Transition Model

The step length and the heading variation within one step constitute the control vector *u* of inertial pedestrian occupancy grid-based FastSLAM. Then, the pedestrian’s pose can be achieved recursively as follows:

(23) $$\begin{array}{cc} s_{t} & =s_{t-1}+u_{t}\\ \left[\begin{array}{c} p_{x,t}\\ p_{y,t}\\ \psi_{t} \end{array}\right] & =\left[\begin{array}{c} p_{x,t-1}\\ p_{y,t-1}\\ \psi_{t-1} \end{array}\right]+\left[\begin{array}{c} d_{t}\cos\left(\psi_{t-1}\right)\\ d_{t}\sin\left(\psi_{t-1}\right)\\ \Delta \psi_{t} \end{array}\right] \end{array}$$

for which $p_{x,t}$ and $p_{y,t}$ are the pedestrian’s position at time *t*, $\psi_{t}$ is the pedestrian’s heading at time *t*. That is, a new pose could be generated based on the state transition model of pedestrian Equation (23), from the pose of the *m*-th particle at time step t−1, the current control input $u_{t}$ and the characteristics of the noise on $u_{t}$. Here, we assume that the step length estimation noise and heading change estimation noise both obey a Gaussian distribution. The result is that the particles are distributed according to Equation (17).

#### 3.2.2. Virtual Range Sensor

In order to perceive the accessibility of grids in the environment, we construct a virtual range sensor using inertial information. This sensor can be seen as a laser range sensor with a very short sensing range. Meanwhile, compared to the laser sensor, which perceives the distance between the obstacle and the sensor, the virtual range sensor perceives the distance between the pedestrian and the accessible grid. The virtual distance sensor measures the distance from the grid where the pedestrian is currently located to the nearest accessible grid. Obviously, the grid which the pedestrian located is the accessible area. Thus, the sensor output is always zero.

The occupancy grid mapping algorithm requires a marginalized inverse measurement model, $p(\theta_{i}|s_{1:t},z_{1:t})$, as shown in Equation (19). This probability provides information about the environment conditioned on a measurement caused by this environment. In this paper, a simple ad hoc inverse measurement model is provided based on the assumption that the more times a grid is accessed, the more likely it is to be accessible. We use a beta distribution to characterize the uncertainty of inverse measurement model and its expectation yields:

(24) $$p\left(\theta_{i}|s_{t},z_{t}\right)=E\left[\mathrm{Be}(\alpha_{i}+\alpha_{0},\beta )\right]=\frac{\alpha_{i}+\alpha_{0}}{\alpha_{i}+\alpha_{0}+\beta }$$

where $\alpha_{i}$ is the total times of the *m*-th particle goes through the *i*-th grid. $\alpha_{0}$ is the prior parameter represents the possibility of *i*-th grid being passed through and is selected as 1 typically. β is the prior parameter represents the possibility of the *i*-th grid not being accessed and is selected equal to $\alpha_{0}$ that is, $p(\theta_{i})$ is 0.5.

It can be seen from Equation (24) that $p(\theta_{i}|s_{t},z_{t})$ increases with the increasing of the number of total times of the grid being accessed. The value of $\alpha_{0}$ and β determine the initial probability of the inverse measurement and the probability that the inverse measurement increases when the grid is accessed again.

#### 3.2.3. Occupancy Grid Update

**Figure 6:**
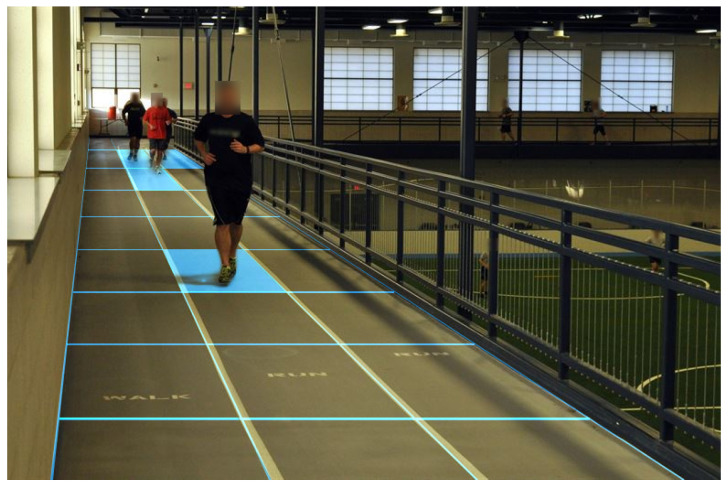
Illustration of the occupancy grids.

The idea of the occupancy grid map is to divide the entire environmental space into a number of discrete grids Θ in equal size, as shown in Figure 6. Each grid $\theta_{i}$ is assigned a probability value $p\left(\theta_{i}\right)\in \left(0,1\right)$, representing the number of grid occupied by the likelihood. In the proposed algorithm, $p(\theta_{i})=1$ means the space of *i*-th grid is accessible for pedestrian and $p(\theta_{i})=0$ means the space of *i*-th grid is intransitable. The size of the grid is an important parameter in the occupancy grid mapping algorithm. In the laser range sensor-based mapping method, the laser range sensor pixel size is chosen as the grid edge length which allows the measurement of obstacles to be continuous and without overlap.

In the proposed method, it is difficult to guarantee non-overlapping observations of the approachable grid due to the inconsistent step size of pedestrian movements. However, it is still possible to guarantee the continuity of the observation of the accessible grids by limiting the edge length of the grids to no less than the minimum step length. Excessively large grid edge lengths result in uncorrected errors in the position estimation of pedestrians moving within one grid. The grid edge length is therefore chosen as the maximum step length, which ensures continuity of observation of the approachable grid and allows the pedestrian positioning error to be corrected in a smaller number of steps.

As opposed to the original occupancy grid update that estimates the probability distribution of obstacle position, the inertial occupancy grid update estimated the probability distribution of accessible space, such as the corridor and rooms in the indoor environment, shown as the blue grid in Figure 6.

The inertial pedestrian occupancy grid-based FastSLAM implements the occupancy grid map update using Equation (21), where the first term is under beta distribution as shown in Equation (24).

#### 3.2.4. Weight Update and Resampling

The weight associated with each particle *m* is updated using the formula for a FastSLAM weight update Equation (22), which for the importance density function in inertial pedestrian occupancy grid-based FastSLAM resolves to:

(25) $$w_{t}^{\left[m\right]}=\eta \cdot w_{t-1}^{\left[m\right]}\cdot {\left\{\frac{\alpha_{i}+\alpha_{0}}{\alpha_{i}+\alpha_{0}+\beta }\right\}}^{\left[m\right]}$$

where η is the normalize factor. Frequent resampling leads to a loss of particle diversity, making it difficult for particles to form loops. To avoid the loss of particle diversity due to frequent resampling, the degree of particle diversity is assessed before resampling:

(26) $$D=\frac{1}{\sum_{m=1}^{N}w_{t}^{\left[m\right]}}$$

The particle set is resampled for importance only if *D* is too large.

## 4. Results

**Figure 7:**
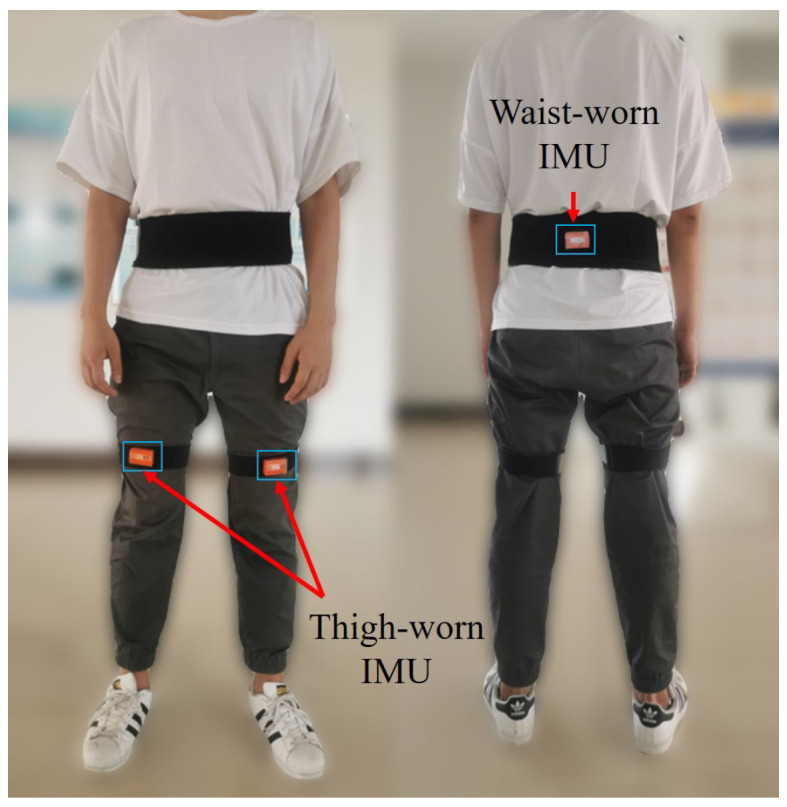
Illustration of the trinal-IMU pedestrian navigation system sensors deployment.

To validate the application effect of the proposed inertial pedestrian occupancy grid-based FastSLAM, several different experiments were conducted on different testers. As illustrated in Figure 7, three MTw (Xsens) inertial measurement units were attached to the pedestrian’s thighs and waist. The six-axis inertial data of each sensor were output at a sampling frequency of 100 Hz. The inertial information was recorded in a laptop during the test and post-processed by the software developed in MATLAB environment according to the algorithm presented above.

### 4.1. Validation of the Step Length Estimation for Drastic Motions

**Figure 8:**
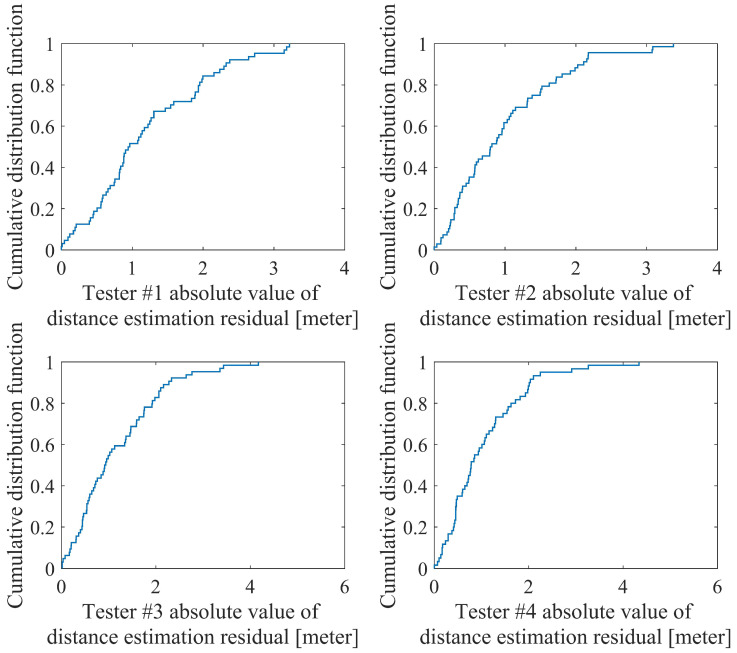
The cumulative distribution function of absolute value of distance estimation residual.

The first test was designed to verify whether the proposed step length estimation method was suitable enough for regular walking, running, and sprint motions. Four testers, including two males and females, were asked to walk, run, and sprint along a 55.8 m straight track at least 12 times for each motion. After calculating the step length estimation parameters for each tester, the cumulative distribution function of the absolute value of the distance estimation residual is shown in Figure 8. The root mean square error of each step length estimation in this process is shown in Table 1. In Figure 8, 93.8%, 95.6%, 92.2%, and 95.0% of the four pedestrian’s different motions absolute distance estimation residuals are in the range of [–3 m,3 m] (5% of 55.8 m). In addition, it can be seen from Table 1 that the proposed method has better adaptability to higher speed motion under the premise of obtaining average accuracy under low-speed motion and the characteristics of the distribution of the residuals of the step length estimates for each step are generally consistent with a Gaussian distribution. Therefore, in the state transition Equation (23), the Gaussian distribution can be used to describe the noise characteristics of the step length control vector.

These results indicate that it is applicable to utilize the proposed method to estimate the step length both in walking, running, and sprint motions. Once the parameters are determined in the initialization procedure, for example, walking along a track in different motions, the step length can be estimated in real-time.

### 4.2. Evaluation of the Occupancy Grid-Based FastSLAM for Inertial Pedestrian Navigation

**Figure 9:**
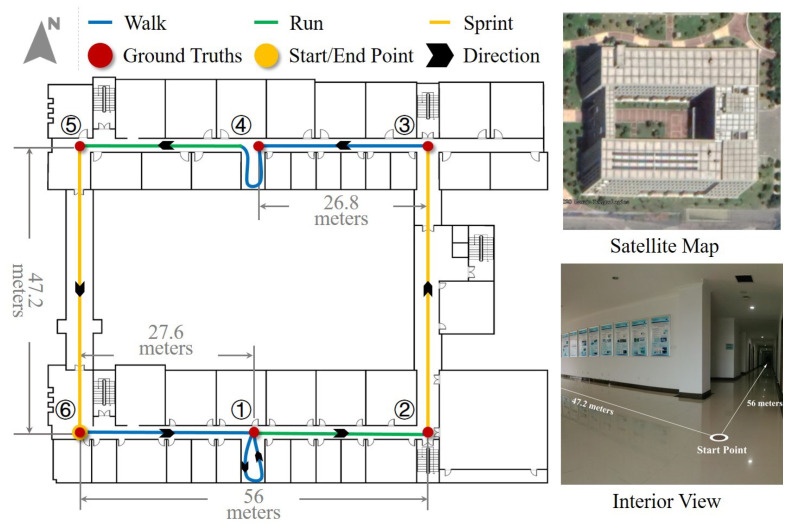
The occupancy grid-based FastSLAM experiment environment and designed path.

In order to evaluate the occupancy grid-based FastSLAM for inertial pedestrian navigation algorithm, the indoor experiments were conducted in a rectangular area of the building in Nanjing University of Aeronautics and Astronautics. As shown in Figure 9, the route is 1083.5 m long and includes two-room walking path. The starting position and ending position are the same point. We select six turners with prior position information as ground truth points to assess the pedestrian position error. Testers were asked to move along the pre-established route five laps in hybrid motion modes, including normal walking, running, and sprint, as illustrated in Figure 9. In this test, 500 particles are selected, and the edge length of the grid is 2 m.

**Figure 10:**
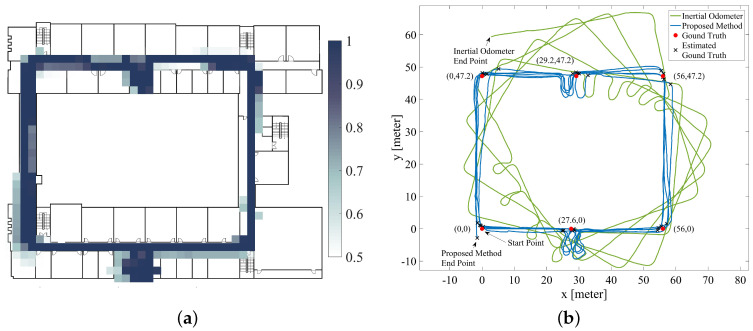
(**a**) inertial occupancy grid-based FastSLAM map; (**b**) the trajectory of inertial pedestrian odometer and the trajectory of inertial pedestrian odometer integrated with occupancy grid-based FastSLAM.

Figure 10a presents the estimated occupancy gird map using the proposed method. The colored grid is the normalized result of the sum of the probability maps carried by all the particles, with white indicating the probability that the grid is passable is 0.5, and dark blue indicating the probability that the grid is passable is 1. Figure 10b compares the estimated trajectory from the inertial odometer (blue line) and the estimated trajectory from the proposed SLAM method (green line). It should be noted that the green curve is the expected distribution of all particles at each moment, rather than the final posterior distribution of all pedestrian pose sequences as shown by most SLAM methods. In other words, once time *t* is experienced, the expected position in the graph at time *t* will not change due to the subsequent optimization process. The reason we do this is that in most scenarios the user is most concerned with the accuracy of navigation at the moment. The expectation of posterior distribution of posture at all final moments after optimization has a smaller error than the above method, which may mislead the researchers’ evaluation of the navigation system.

**Figure 11:**
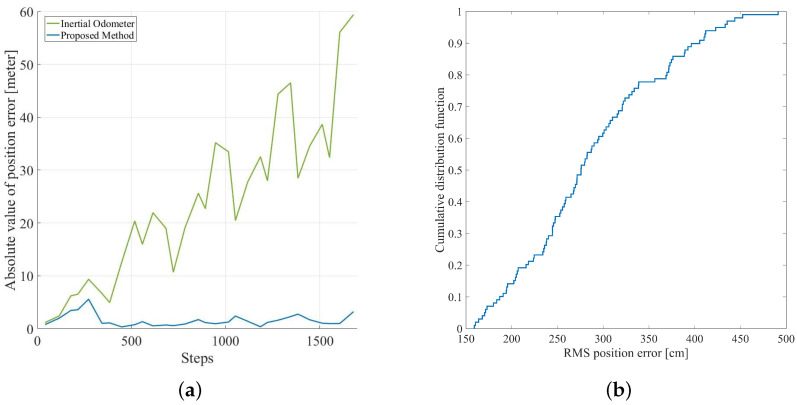
(**a**) the absolute value of position error of inertial pedestrian odometer and inertial pedestrian odometer integrated with occupancy grid-based FastSLAM; (**b**) the RMS position error cumulative distribution of inertial pedestrian odometer integrated with occupancy grid-based FastSLAM.

The tester was asked to go through some ground truths mentioned above; during the test, the tester recorded the number of steps taken before the moment through ground truths. The estimation of the position of these ground truths is shown in the black cross in the figure; meanwhile, the red circles indicate the true position of the ground truths. Figure 11a illustrates some of the main characteristics of the positioning error and the number of times pedestrian passes the ground truths, in which the positioning error of the inertial odometer (green line) accumulated over time and the positioning error of proposed inertial occupancy gird-based FastSLAM is always kept at a low level. The final absolute return position error is 59.41 m and 3.22 m, respectively, and the relative error is 5% and 0.3%, respectively.

In order to eliminate the influence of the Monte Carlo particle sampling randomness to the location result, we have processed the above data 100 times, and the cumulative distribution of the RMS position error between the trajectory and the ground truths are shown in Figure 11b. It can be seen from the graph that the probability of average error less than 4 m is 89.9%.

The second experimental scenario is a random walk in multiple rooms. The experimental route consisted of multiple turns, which posed a challenge for heading estimation. The tester was asked to walk around the multiple rooms randomly, and return to the starting point after every certain time. We used the starting point as ground truth to evaluate the positioning accuracy of the system. The parameter settings of the system remained the same as in the previous experiment.

**Figure 12:**
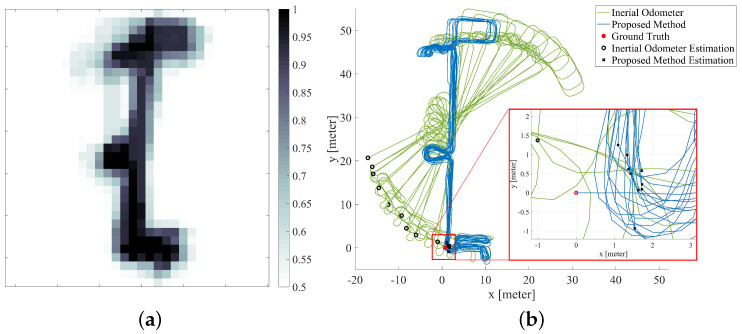
(**a**) inertial occupancy grid-based FastSLAM map; (**b**) the trajectory of inertial pedestrian odometer and the trajectory of inertial pedestrian odometer integrated with occupancy grid-based FastSLAM.

**Figure 13:**
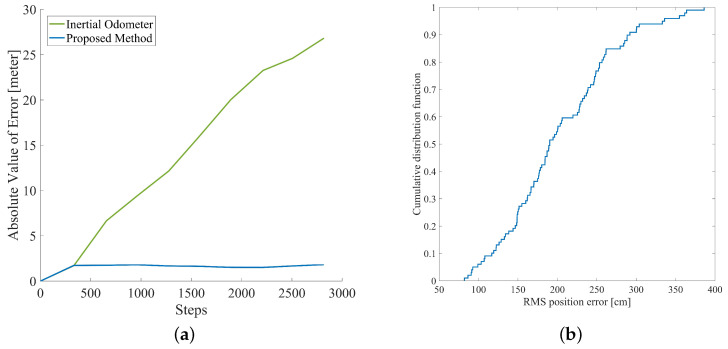
(**a**) the absolute value of position error of inertial pedestrian odometer and inertial pedestrian odometer integrated with occupancy grid-based FastSLAM; (**b**) the RMS position error cumulative distribution of inertial pedestrian odometer integrated with occupancy grid-based FastSLAM.

Figure 12a shows the map constructed by the proposed method based on the testers’ movements. Figure 12b compares the estimates of pedestrian trajectories from the inertial odometer and the proposed method. The green line shows the positioning results of the inertial odometer and the blue line shows the positioning results of the proposed method. It can be seen that the heading of the inertial odometer gradually diverges, while the heading estimate of the proposed method is very stable. The red point is the starting point of navigation and also the ground-truth of the path, the black circle indicates the ground truth estimate of the inertial odometer, and the black cross indicates the ground truth estimate of the proposed method. The variation of the RMS ground truth estimation error over time is shown in Figure 13a, where the green line is the absolute value of the ground truth estimation error of the inertial odometer, and the proposed method corresponds to the blue line. It can be seen that the positioning error of the proposed method does not increase with time, effectively eliminating the cumulative error of the inertial odometer.

Similarly, in order to eliminate the influence of the randomness of Monte Carlo particle sampling on the localization results, we processed this data set 100 times, and the cumulative distribution function of the RMS position error is shown in Figure 13b; the proposed method has an error of less than 3.36 m at a 95.96% confidence interval.

## 5. Discussion

The main purpose of this study is to develop a multi-IMU inertial pedestrian odometer integrating with the occupancy grid-base FastSLAM approach to improve the performance of the inertial pedestrian navigation system on drastic motions. The traditional PDR based inertial pedestrian navigation system has advantages including no additional infrastructure needed and no radio frequency interference but has disadvantages including the inaccurate step length estimation on drastic motions such as running and sprint, and unbounded error on heading estimation. We suggested a simple step detection and segment approach by detecting the zero-crossing of the pitch of thighs. Meanwhile, a step length estimation method for drastic motion based on the pdf of the waist-worn horizontal acceleration norm is proposed. These two methods together constitute an inertial pedestrian odometer adapted to drastic motions. The experiments on different testers on different speed motions indicate that the proposed inertial pedestrian odometer can provide the step length estimation with the same precision as the slow one when the pedestrian is moving fast. Moreover, we extended the occupancy grid-based FastSLAM algorithm to the inertial navigation system. By constructing a virtual range observer, the simultaneous positioning and mapping of pedestrians can be realized by relying on inertial odometer without any additional sensors. The experiment results indicate that the proposed inertial occupancy grid-based FastSLAM can accurately construct the map of the passable area, and the system can effectively restrain the course error divergence of pedestrian inertia odometer. This method can provide long-term and high-precision pedestrian positioning information without increasing the cost, which is of great significance for the practical application of pedestrian inertial navigation.

The proposed method can improve the long-term accuracy of inertial pedestrian navigation system. However, there are still some limitations in this study. Firstly, the inertial occupancy grid-based FastSLAM utilizes the historic trajectory information to correct the positioning and heading error which means that the position and heading errors are corrected only when the pedestrian revisits the area they have already visited. Secondly, the determination of the initial position is not considered in this study. We focus on solving the issue of accumulative error rather than the initial position. Nevertheless, according to the basic principles of inertial navigation, the initial position error will be gradually coupled to the attitude estimation. On the one hand, it will lead to an increase of course estimation error; on the other hand, it will lead to inaccurate waist attitude estimation, which will affect the step length estimation, which limits the application of the system in larger areas.

For the first limitation, we can use the historical trajectory of multiple people to correct map estimation and user location estimation by studying the method of multi-pedestrian collaborative SLAM. For the second limitation, the combination of GPS and the inertial system can be used to obtain the absolute position of pedestrians through the satellite navigation system before they enter the blocked area of the satellite.

## 6. Conclusions

In this paper, we propose a triple-node IMU inertial pedestrian odometer which improves the accuracy of inertial pedestrian localization in dealing with drastic motions. Compared to the traditional PDR system, the proposed approach can provide the step length estimation with the same precision as normal walking when the pedestrian is running or sprinting. Moreover, we extended the occupancy grid-based FastSLAM, which is commonly used in a vision-based navigation system, to the inertial pedestrian navigation by constructing a virtual range sensor to precept the external environment. The combination of these two methods could estimate step length accurately and eliminate the accumulated positioning error of the inertial pedestrian odometer. Less than 5 m positioning accuracy in a single-story building of 2643.2 $m^{2}$ is achieved. In future work, we plan to extend the single inertial occupancy grid-based FastSLAM method to the multi-pedestrian collaborative SLAM method.

## Figures and Tables

**Table 1:** Step length estimation RMS error of three models.

Tester	Average Velocity [m/s]	Linear Method [5]		Nonlinear Method [6]		Proposed Method	
		Absolute Error RMS [meter]	Relative Error RMS	Absolute Error RMS [meter]	Relative Error RMS	Absolute Error RMS [meter]	Relative Error RMS
	1.57	0.90	1.61%	1.35	2.42%	2.00	3.58%
#1	2.66	4.80	8.57%	4.04	7.22%	0.80	1.42%
	3.48	4.88	8.72%	8.40	15.00%	0.53	0.94%
	1.42	0.83	1.49%	1.72	3.06%	1.07	1.91%
#2	2.90	4.66	8.32%	2.77	4.95%	1.20	2.15%
	3.92	5.41	9.65%	8.54	15.24%	1.36	2.42%
	1.24	1.20	2.14%	2.84	5.07%	1.20	2.14%
#3	1.95	2.44	4.36%	9.10	16.26%	2.39	4.27%
	2.34	3.39	4.27%	1.87	3.33%	1.99	3.55%
	1.23	0.98	1.74%	6.08	10.86%	1.07	1.91%
#4	2.06	4.29	7.66%	13.06	23.31%	0.01	0.03%
	2.56	6.62	11.83%	10.53	18.81%	0.03	0.06%
